# Neuralgia and Other Disorders Caused by Uterine Disease

**Published:** 1878-12-20

**Authors:** D. N. Kinsman

**Affiliations:** Prof. of Practice of Medicine in Columbus Medical College, Columbus, Ohio


### NEURALGIA AND OTHER DISORDERS CAUSED BY UTERINE DISEASE.

BY D. N. KINSMAN, M. D.,
Prof. of Practice of Medicine in Columbus Medical College, Columbus, Ohio.

CASE 1.—A., married, aged 30, mother of three children, eldest five years, the youngest died at $4\frac{1}{2}$ months would at the time of taking these notes, have been 6 months old. The patient has suffered from dysmenorrhea ever since menstrual life begun.

Since the birth of her first child five years ago, the suffering at her catmenial periods has been much less, but she has had irregular attacks of pain and sickness occuring as the result of fatigue, emotion, exposure to cold, indigestion, etc.

She is pale, emaciated, nervous, and lives in constant apprehension of her "spells." Concerning these spells, they are introduced by pain and dragging in the pelvis and become constantly intensified. The pain then radiates to the bladder, causing dysuria, to the abdomen and stomach when there is cramping pain, into the hips and down the anterior cervical nerves. These pains continue increasing in severity for several hours; with the cramps in the stomach there is intense pain in the inter-scapular region of the spine. These attacks are sometimes sudden, rousing her from sleep. During these paroxysms the stomach can be felt to contract and become hard as the result of the contracting of its coats.

This is accompanied with abundant eructations of an oderless gas succeeded by nausea and vomiting. The matters vomited, are first, the contents of the stomach, followed by mucus stained with bile, afterwards the drinks which are given will be rejected, with patches of grass green color, looking like pounded grass. When once this condition is established it seems to have little tendency to subside, and continues for weeks at a time. There is fever of low grade, gastric tenderness, dry tongue, and feeble pulse. The spinal column is tender along its whole extent. The head painful at the vertex and occiput—during the greatest severity of the attacks there is delirium, photophobia and intolerance of sound. This patient "has suffered many things of doctors, getting no better but rather worse." Was advised by the most eminent of those living in her region. She had been treated for gastritis, by means of blisters to the epigastrium, hydro-cyanic acid, morphia, bismuth, id omne genus; for spinal irritations for which the spine has been kept blistered, or raw with iodine, or pustulated with croton oil for weeks at a time; for trouble with the liver by murcurials to the extent of salivation; for dyspepsia, by bark, mineral acids, bismuth, pepsin, strychnia, all without avail. These "spells" were treated by means of opiates, of which drachm doses of laudanum repeated hourly were necessary, to this was added chloroform by inhalation, and I have given an ounce of the former and a pound of the latter in eight hours.

I never have witnessed more dreadful suffering than this poor woman suffered. Originally vivatious, cheerful and hopeful, she became despondent and full of apprehension for the future.

This case now detailed occurred twelve years ago, when special works on gynecology in English were not common as they are now. Scanzoni, Churchill and

Meigs were the only authorities at my command. At that time Thomas' first edition was published and gave light.

Upon examination the uterus was found excessively tender to the touch, or patulous, a string of mucus clear and glairy depending from it, tenderness in ovaries. The introduction of the probe caused intense pain both in the cervix and at the fundus; this enabled me to diagnose cervical and corporeal endometritis but caused a new attack for the patient which did not subside for more than a week.

Treatment adopted consisted of irrigations of hot water applied to uterus through the vagina, scarifications of the cervex uteri; and the swabbing of the uterine cavity with carbolic acid, full strength of the melted crystals—alternated with the use of iodine used in the same manner. Under this treatment patient recovered, and 18 months ago gave birth to a fourth child, since which there has been no return of former trouble. She can walk miles, has increased in flesh and is in as good health as ordinary women.

Case 2.—Mrs. B., age 25, married for two years. Has been a sufferer from neuralgia for many years confined to the branches of trigeminus. Since puberty has suffered occasionally from dysmenorrhea of severe character, at other times there has been comparatively little suffering. Quanity of menstrual fluid judged to be normal, the pain occurs at the begining of the epoch and subsides on second day. During the last year her suffering has taken an entirely different turn; no longer in the face, it has become located in the left hip and along the left sciatic nerve. There is an iscrease of suffering at each menstrual epoch, and there is slight leucorrhea in the intervals. There is a slight tenderness of the spinal column in the sacral and lower dorsal region, no points of tenderness in the course of the sciatic nerve. Under these circumstances as she had been long treated by means of tonics, I deemed it proper to investigate the condition of the uterus.

Upon conjoined manipulation the body of uterus was found tender, and there is anteflexion; the introduction of the sound, which was accomplished with no little difficulty, enabled me by gentle blows upon the upper portion of the uterine cavity to evoke the sciatic twinges in the hip as well as cause very decided evidences of trouble with the endometrium. The os uteri was exceedingly small and tortuous.

Treatment: dilution, by means of laminaria tents, with application of carbolic acid applied to the uterine cavity by means of a cotton swab.

Treatment was continued two months at intervals of a week, when she became pregnant, and further treatment was abandoned. She improved constantly from the first application. Since her pregnancy there has been no further trouble.

Case 3.—Miss —, teacher, age 22. Has suffered from dysmenorrhea ever since puberty. Quantity scanty, pain during the whole of the menstrual period, intense pain over the region of the ovaries. During the inter-menstrual period, suffers continously with pain in the cervix and occipital region of head, bowels constipated, appetite capricious. Uterine examination reveals tenderness of the whole womb. The probe with difficulty enters the os internum. There is but little leucorrhea. The treatment was introduced by using a laminaria tent. At the expiration of eight hours I was called in great haste to see her, because she was suffering great pain. I found her vomiting, pulse hard and frequent, tender over lower portion of the abdomen.

Attributing the disturbance to the tent I attempted to withdraw it, by the string attached, but found I was unable to do it, and that the uterus descended when I made traction. I then seized it with a strong pair of dressing forceps, supporting the lips of the cervix with my fingers, by making strong traction I

succeeded in withdrawing the tent. On examining the tent I found the portion in the cavity of the womb and that in the cavity of the cervix had both dilated to there full capacity, ¼ of an inch, while the portion surrounded by the os interum had been unable to overcome its tension and had remained constricted to a trifle over one twelveth of an inch, and the shoulder above this portion must have ruptured the constricting fibres in withdrawing it. There was a small escape of blood. The case was alarming, morphia was administered hypodermically, hot poultices were ordered to the abdomen; this treatment was continued with addition of aconite the second day. In ten days the disturbance subsided, the patient returned to her home, no further treatment being deemed advisable. I saw the patient three months after when she reported herself much better in every respect than she had ever been before, and expressed great gratitude to me for curing her.

It is needless for me to say that her thanks seemed almost mockery to me when I remembered how near she came to losing her life from the treatment, and how grateful I was that I was spared the mortification of adding her name to the list of those who have died from the use of the tent. The cure has remained permanent now three years. Why?

Case 4.—May 6th, 1877. I was called to see Mrs. M., mother of three children. Health has been as good as the average until three months ago. In December 1876 she aborted, at what month not learned, from this she recovered fairly, since that time she has never menstruated. In February at a date now unknown she began to suffer pain in the stomach whenever she took food even the blandest. The pain began very soon after eating, and continued for some time when she would vomit and for a time have relief. The matters vomited would at times be merely tinge with blood, at others, at the close of the act of vomiting there would be an ounce or more of fresh looking blood thrown up. She is not strikingly emaciated, on the other hand has a rather good color which shows she is fairly nourished notwithstanding the vomiting and hæmatemesis. There is no pain on pressure, there is no tumor to be felt, there is no positive evidence of stomachal disease to be derived from palpation. The bowels never discharge tarry and black stools, which shows little more blood escapes than is vomited. Keeping in mind a maxim long ago adopted by myself, never to neglect to examine the uterus in an obscure case in a woman, I next proceeded to do this. When I found this organ enlarged and showing every evidence of pregnancy. I then announced she was three months pregnant, and that another month would relieve her of her trouble when the uterus rose from the pelvic cavity. Time verified my prognosis for she was relieved of her vomiting in June and in October of her baby.

In the first case detailed, we have every symptom of gastritis of a severe type, and yet their frequent repetition ought to have excluded this disease, for on the subsidence of these after repeated acute attacks no symptoms of the chronic form of the disease remained which must inevitably have been the case had there been structural lesion of the membrane of the stomach. Indigestion which seemed to be the exciting cause of the distress in the stomach and vomiting, was a secondary phenomenon, and bore the same relation to the uterine disturbances that vomiting does to migraine, being secondary, a consequence of the central disturbanee and not the cause, a fact first pointed out I think by Dr. Samuel Wilkes.

This relation would have been fairly inferred from the pessistent nausea and vomiting lasting days and weeks without rise of temperture, and in spite of all treatment addrsssed to the condition of the stomach.

And yet the flatulence, vomiting succeeding the eating of food, the pain in the stomach and in the interscapular region of the spine, followed by epigastric tenderness, dry tongue, and feeble pulse and delirium, were sufficient for a long time to cloud the diagnosis in the minds of those who were not awake to the range of influence a diseased uterus might exert.

In the second case the mother of the patient had suffered years from chronic rhuematism, and undoubtedly the trouble in the trigeminus was of rheumatic character.

The patient was married, her new relation in some way had decided the localization of the trouble in the hip—or rather in the left sciatic nerve.

That the sciatica had a very intimate relation with the condition of the endometrium was shown in this case by the setting up of the neuralgia by the contact of the probe in the examination. In the case first detailed the relation of the uterus to the stomachal trouble was distinctly shown in the attack which followed immediately upon the examination of the cavity of the uterus with the probe. And this immediate relation of the uterus when it can be made out by an examination, is one of the strongest possible points in making a diagnosis.

Case four, places in the very strongest light the necessity of a complete and thorough examination of the generative organs in a female, whose case is involved in any obscurity. This patient had been seen by six physicians besides myself, all of whom with one exception had come to the conclusion there was gastric ulcer.

This physician suspected there might be pregnancy but made no examination, and he treated the case as one of gastric ulcer. The recent abortion, the non-return of menstruation subsequently seemed to exclude the possibility of pregnancy, and all symptoms seemed to indicate gastric ulcer. When this case was described to the Columbus Pathological Society, suppressing the fact developed by vaginal examination, it was thought to be gastric ulcer.

Case third exemplifies the most common manifestation of pain as the reflex of uterine disease. This is almost always present and showed direct attention to the uterus. The uneasiness in the cervix may be simply a burning or an intense bursting pain, and it is increased by anything which increases the uterine disturbance.

This case also illustrates how suddenly threatening symptoms may arise from the use of tents, and the caution with which they should be used.

In my treatment of uterine diseases I have used for the last ten years carbolic acid crystals, liquified by heat, as a local application, both to the cervix and fundus. It is a local anæsthetic and is an antidote thus to its own action, in a short time after its application all pain ceases. It is as powerful as argenti nitratis, is less painful, and there is no tendency to contraction of the parts, such as is constantly seen upon the use of the nitrate. This process of contraction hides the diseased part—and I have known many cases discharged cured, who in a few weeks returned as bad as ever. Latterly I have used fuming nitric acid, as recommended by Athill and others, by a method made known to me by my friend Dr. N. R. Coleman, of Columbus, which I find possesses many advantages over any method I have hitherto used.

A piece of white wax smoothed down to the requisite size to enter the cervical cavity and given the proper curve, is dipped into the acid; a sufficient quantity adheres, for the purpose to which it is to be applied. The wax bougie is grasped with a pair of dressing forceps and passed into the cervix; applied in this manner there is no excess of acid to run over adjacent parts, while there is enough to act as a caustic or alterant as you please to term it.—*Obstetric Gazette.*

